# Geographical Relationships between Long-Tailed Goral (*Naemorhedus caudatus*) Populations Based on Gut Microbiome Analysis

**DOI:** 10.3390/microorganisms9092002

**Published:** 2021-09-21

**Authors:** Chang Eon Park, Bum-Joon Cho, Min-Ji Kim, Hee Cheon Park, Jae-Ho Shin

**Affiliations:** 1 Department of Applied Biosciences, Kyungpook National University, Daegu 41566, Korea; aeonrapt@knu.ac.kr (C.E.P.); tbd01188@gmail.com (M.-J.K.); 2 Institute of Ornithology, Association of Ex-Situ Conservation, Daegu 41541, Korea; cranesave@naver.com; 3 Wildlife Union, Donghae 25802, Korea; hl2xsg@hanmail.net

**Keywords:** *Naemorhedus caudatus*, long-tailed goral, geographical relationship, gut microbiome, 16s rRNA sequencing, endangered species, ruminant, network analysis

## Abstract

The long-tailed goral (*Naemorhedus caudatus*) is an endangered species found in the mountains of eastern and northern Asia. Its populations have declined for various reasons, and this species has been designated as legally protected in South Korea. Although various ecological studies have been conducted on long-tailed gorals, none have investigated the gut microbiome until now. In the present study, we compared the diversity and composition of the gut microbiome of seven populations of Korean long-tailed gorals. By analyzing the gut microbiome composition for each regional population, it was found that four phyla—Firmicutes, Actinobacteriota, Bacteroidota, and Proteobacteria—were the most dominant in all regions on average. The alpha diversity of the gut microbiome of the goral population in the northern regions was high, while that in the southern regions was low. Through the analysis of beta diversity, the seven long-tailed goral populations have been divided into three groups: the Seoraksan population, the Samcheock population, and the Wangpicheon population. It was possible to confirm the regional migration of the animals using the gut microbiome based on the site-relational network analysis. It was found that the most stable population of long-tailed gorals in Korea was the Seoraksan population, and the closely related groups were the Samcheok and Wangpicheon populations, respectively. Wangpicheon appeared to be a major point of dispersal in the migration route of Korean long-tailed gorals.

## 1. Introduction

The long-tailed goral (*Naemorhedus caudatus*) is a vulnerable species distributed in the eastern and northern Asian mountains, including Russia, China, and Korea [1,2]. In South Korea, long-tailed gorals have experienced a genetic bottleneck effect by the rapid decrease in their population size as thousands of gorals died due to heavy snowfalls and hunting in 1964 and 1965. Their populations declined sharply due to poaching, habitat destruction, and abnormal climate conditions, resulting in decreased genetic diversity [3,4]. As a result, the long-tailed gorals were designated as an endangered species by the Ministry of the Environment, and as a natural monument by the Cultural Heritage Administration. In addition, various studies are being conducted to prevent extinction, but no research has been investigating the gut microbiome in this species yet.

The animal gut microbiome is determined by its host [5,6,7] and can reflect geographic features that characterize the host itself [8], which contributes to understanding the animal’s ecology. Studying the animal gut microbiome by analyzing variable data allows researchers to overcome the limitations of a biogeographic perspective. Previous research methods mainly studied haplotypes by region using partial mitochondrial DNA [3]. This type of study can reveal regional differences, but they are mainly used for phylogenetic research, and they cannot adequately explain the relationships between regions. The majority of other studies related to long-tailed gorals are based on field research. In the field, photos are taken using a camera trap to identify individuals [9], or a geographic information system is used to analyze habitat use [10]. Still, field research cannot address issues such as the regional relationships between goral populations in different regions.

However, the gut microbiome of animals presents a species-specific pattern and various information can be obtained from it, ranging from the species to the genus, family, order, class, and phylum, and each taxon is composed of various strains. Therefore, it is possible to explain long-tailed gorals’ regional characteristics and relationships based on the wide range of microbial information available. Most studies describe long-tailed gorals as a resident species [11]. However, the absence of migration between populations cannot explain the presence of long-tailed gorals in new areas. Therefore, the purpose of this study was to analyze the diversity and composition of the gut microbiome among different populations, based on the regional relationship of long-tailed goral populations and to understand their migration routes and patterns from the microbial ecology perspective.

## 2. Materials and Methods

### 2.1. Sample Collection

**Figure 1:**
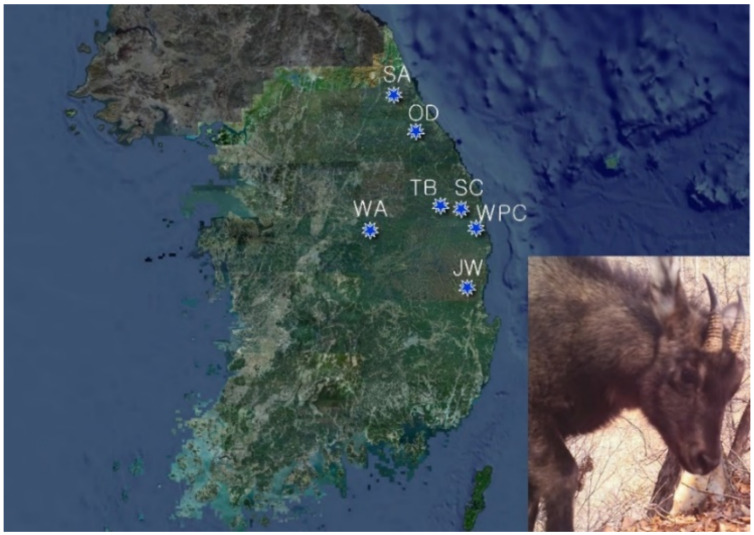
Map of the seven study sites in South Korea, where fecal samples were collected, and photo of the long-tailed goral. Abbreviations: SA = Seoraksan National Park (*n* = 24); OD = Odaesan National Park (*n* = 8); WA = Woraksan National Park (*n* = 6); TB = Taebaeksan National Park (*n* = 3); SC = Samcheok Area (*n* = 27); WPC = Wangpicheon Conservation Area (*n* = 14); JW = Juwangsan National Park (*n* = 4). Map generated from QGIS 3.10.12 with VWORLD map.

Long-tailed goral fecal samples were collected from six conservation sites and one nonconservation site. The former are the Seoraksan National Park, Odaesan National Park, Woraksan National Park, Taebaeksan National Park, Juwangsan National Park, and the Wangpicheon Conservation Area, and the nonconservation site is the Samcheok Area (Figure 1). To avoid soil contamination, only fresh feces from the upper layers were selected for sampling. After collection, samples in sterile low-density polyethylene containers were sent to the laboratory and stored at −80 °C until DNA was extracted.

### 2.2. Microbiota Analysis

Total DNA was extracted from 250 mg of homogenized fecal solution with the Power Fecal DNA Isolation Kit (MoBio Laboratories Inc., Carlsbad, CA, USA) following the manufacturer’s protocol. The extracted metagenomic DNA was used as template for gene amplification of the V4–V5 region of the 16S ribosomal ribonucleic acid (rRNA). The bacterial 16S ribosomal RNA gene sequence-biased primers 515F (5′-GTGCCAGCMGCCGCGG-3′) and 907R (5′-CCGTCAATTCMTTTRAGTTT-3′) were used to amplify polymerase chain reaction fragments that were 420 base pairs in length [12]. Each sequencing sample was loaded on an Ion 318 Chip Kit v2 BC (Thermo Fisher Scientific, Waltham, MA, USA) and sequenced on an Ion Torrent Personal Genome Machine (PGM) (Thermo Fisher Scientific, Waltham, MA, USA) for 1250 flows using an Ion PGM Hi Q Sequencing Kit (Thermo Fisher Scientific, Waltham, MA, USA) [13]. The Ion Torrent PGM was used along with the specific pipeline software Torrent Suite v 5.0 (Thermo Fisher Scientific, Waltham, MA, USA) to generate sequence reads, trim adapter sequences, and filter and remove poor-signal profile reads from the sequencing data [14]. The primer-trimmed files were then imported into Quantitative Insights Into Microbial Ecology 2 (QIIME2) v. 2020.11 software [15,16] for microbiota analysis. Statistical and network analyses were performed in R using the phyloseq package [17,18]. Alpha diversity was measured through the Observed, Chao1, ACE, Shannon, and Simpson indices with R software. The beta diversity was calculated through a maximum distance analysis, with a partial least squares discriminant analysis (PLS-DA) using the mixOmics package [19] and Principal Coordinate Analysis with Bray-Curtis distances in R. All other statistical processing was performed in R.

## 3. Results

### 3.1. Biodiversity Patterns

**Figure 2:**
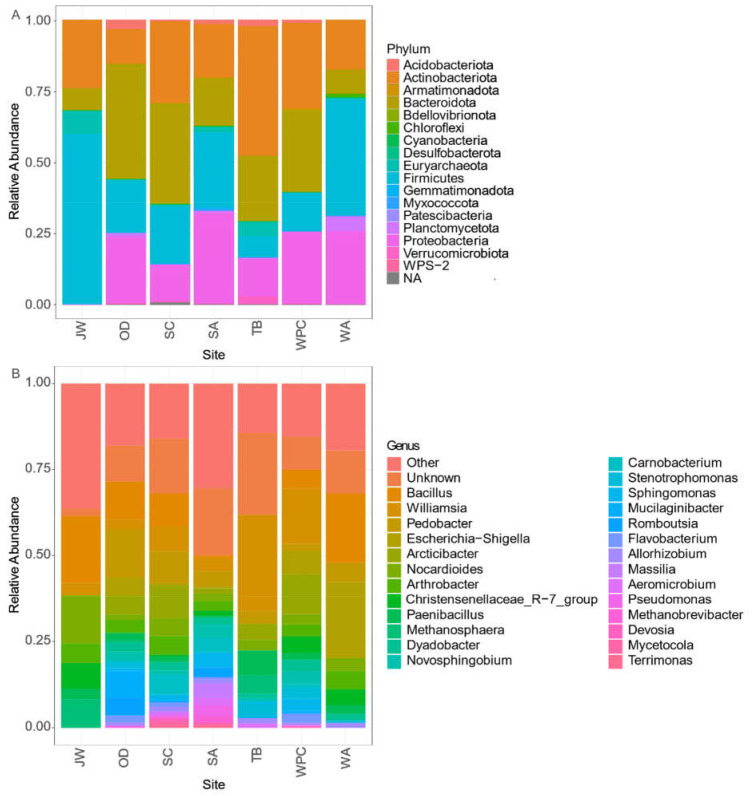
Bar charts representing the relative abundance of the total 16S rRNA sequence, taxonomically classified at the (**A**) phylum level and (**B**) genus level. Abbreviations: JW = Juwangsan National Park; OD = Odaesan National Park; SC = Samcheok Area; SA = Seoraksan National Park; TB = Taebaeksan National Park; WPC = Wangpicheon Conservation Area; WA = Woraksan National Park.

The analysis of the composition and biodiversity of the gut microbiome for each regional population of long-tailed gorals in South Korea revealed that, at the phylum level, the population of Seoraksan National Park presented the most various biodiversity, with 17 phyla, followed by the population of Samcheok Area with 14 phyla, and that of Wangpicheon Conservation Area, with nine phyla. Eight phyla were detected in the Odaesan National Park, Taebaeksan National Park, and Woraksan National Park populations. (Figure 2). Surprisingly, the Juwangsan National Park population contained only four phyla. In the seven populations of long-tailed goral analyzed, the four most dominant phyla detected in the gut microbiome were: Firmicutes, Actinobacteriota, Bacteroidota, and Proteobacteria. The percentage of the gut microbiome varied by region (Table S1). As a result of comparing abundance at the phylum level, there were mainly differences in four phyla (Figure S1).

As for the analysis of the composition and biodiversity of the gut microbiome for each regional population of long-tailed gorals in South Korea on the genus level, the numbers of genus of each population was a very different among the regions. The population of Seoraksan National Park presented the most various biodiversity, with 262 genera, and the population of the Samcheok Area had 136 genera, while the Wangpicheon Conservation Area had 45 genera. Forty-three genera were detected in Odaesan National Park, followed by the population of Taebaeksan National Park, which had 31 genera, and that of Woraksan National Park, with 30 genera. (Figure 2). Surprisingly, the Juwangsan National Park population contained only 17 genera. As a result of comparing abundance at the genus level, there were mainly differences in six genera (Figure S2).

**Figure 3:**
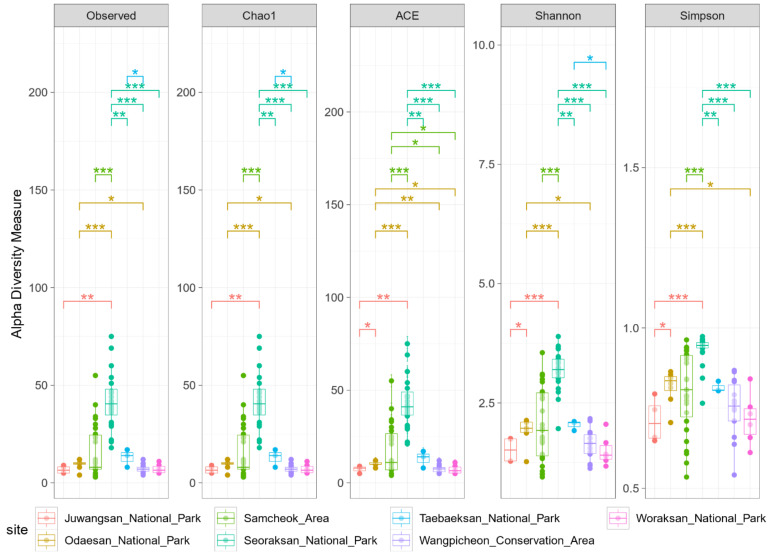
Alpha diversity indices of the gut microbial communities at each sampling site. * *p* < 0.05, ** *p* < 0.01, *** *p* < 0.001.

Alpha diversity indicators, based on sampling sites, showed comparable values for Observed OTU index, Chao1 index, ACE index, the Shannon index, and Simpson’s index. The diversity indices of the gut microbiome in the Seoraksan National Park population were the highest, while those in the Woraksan National Park and Juwangsan National Park populations were the lowest. The analysis of all five indices showed no difference in microbiome diversity among the Juwangsan National Park, Wangpicheon Conservation Area, and Woraksan National Park (Figure 3). The Samcheok Area and Seoraksan National Park have shown a broad variation in all of diversity indices. Surprisingly, Seoraksan showed a significant difference from all other regions.

**Figure 4:**
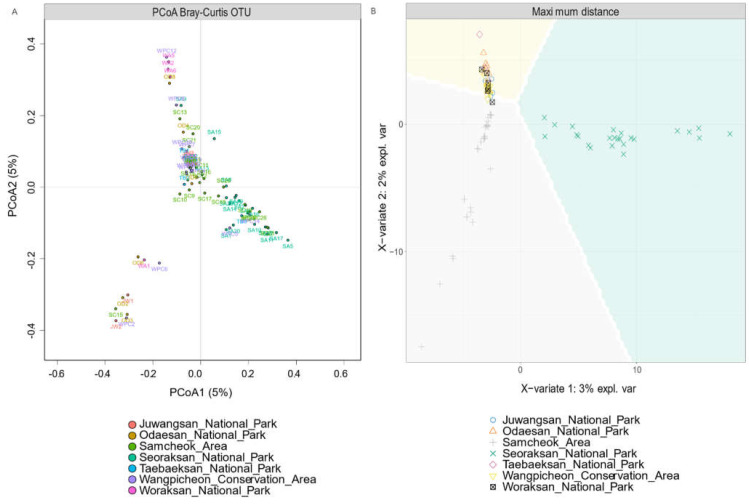
(**A**) Principal Coordinate Analysis using Bray−Curtis distances. Abbreviations: JW = Juwangsan National Park; OD = Odaesan National Park; SC = Samcheok Area; SA = Seoraksan National Park; TB = Taebaeksan National Park; WPC = Wangpicheon Conservation Area; WA = Woraksan National Park. (**B**) Maximum distance analysis of the gut microbial communities between sampling sites. The yellow, green, and gray backgrounds indicate the maximum distance in the Wangpicheon Conservation Area, Seoraksan National Park, and Samcheok Area, respectively.

By the beta diversity analysis using Principal Coordinate Analysis with Bray-Curtis distances, all OTUs were in the 5% range, showing three core groups: Seoraksan National Park and Samcheock Area groups, Wangpicheon Conservation Area group, and other group (Figure 4). Seoraksan National Park, Samcheok Area, Wangpicheon Conservation Area, and Woraksan National Park showed wide dispersion. Most OTUs from Soeraksan and Samcheok were coordinated on the positive PCoA1 axis, and Wangpicheon, Woraksan, Odaesan, Juwangsan and Taebaeksan areas were coordinated on the negative PCoA1 axis. The OTUs of Wangpicheon and Woraksan sites were coordinated at the positive part of the PCoA2 axis and that of Samcheok, Seoraksan, Odaesan, and Juwangsan were located at the negative coordinates.

The total sample was divided into the following three groups in the beta-diversity analysis using PLS-DA with maximum distance: Seoraksan National Park, Samcheok Area, and Wangpicheon Conservation Area (Figure 4). The beta-diversity plots of Seoraksan National Park and Samcheok Area showed a high degree of dispersion, while those of the Wangpicheon Conservation Area had a low degree of dispersion. Except for these three groups, the other plots were located close to the Wangpicheon Conservation Area.

### 3.2. Site-Relational Network Analysis

**Figure 5:**
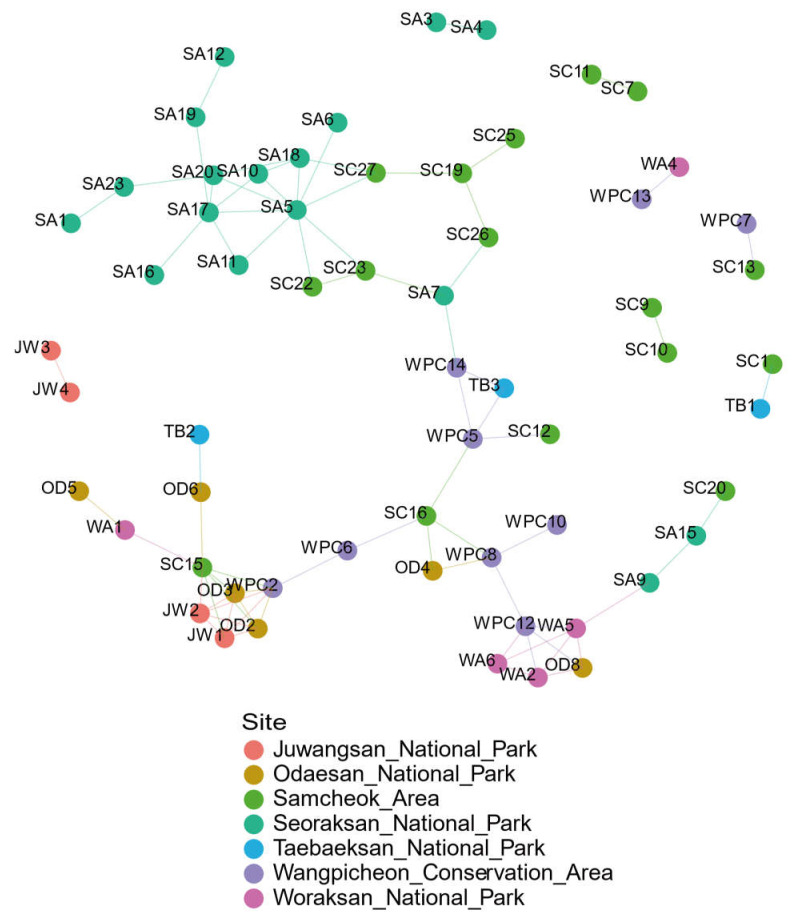
Site-relational network connecting long-tailed goral populations to habitat sites. Abbreviations: JW = Juwangsan National Park; OD = Odaesan National Park; SC = Samcheok Area; SA = Seoraksan National Park; TB = Taebaeksan National Park; WPC = Wangpicheon Conservation Area; WA = Woraksan National Park.

Figure 5 depicts the relative regional relationships between long-tailed goral populations obtained from network analysis. Each network node represents the gut microbiome of long-tailed gorals for a particular region. The color of each line indicates that the microbiome is diffused from the network node of that color. For example, if a green node is connected to another node with a green edge, it means it has spread from that green node. In regional terms, diffusion implies the migration of the animals. The long-tailed gorals of Seoraksan National Park had the closest relationship with those of the Samcheok Area, and there was migration between the two populations. The population of Odaesan National Park was connected to those of the Samcheok Area, Woraksan National Park, Juwangsan National Park, and Taebaeksan National Park instead of being connected to the Seoraksan population, which was located at the nearest distance. The population of Taebaeksan National Park was connected to that of the Wangpicheon Conservation Area. The population of Samcheok Area was connected to those of Seoraksan National Park, Odaesan National Park, Taebaeksan National Park, Wangpicheon Conservation Area, and Woraksan National Park very broadly. The population of Woraksan National Park was connected to those of the Wangpicheon Conservation Area and Odaesan National Park. The population of Wangpicheon Conservation Area was connected to those of Seoraksan National Park, Odaesan National Park, the Samcheok Area, and Juwangsan National Park. Finally, the population of Juwangsan National Park was connected to that of the Samcheok Area. It was found that the goral populations of each region migrated from at least one area.

## 4. Discussion

### 4.1. Biodiversity and Regional Relationships

This is the first research study investigating the regional relationships between long-tailed goral populations and gut microbiome biodiversity. The populations in each region showed different diversity by presenting different percentages of microbiota phyla and genera (Figure 2). In the alpha diversity results, it was found that the gut microbiome of long-tailed gorals was more diverse toward the northern regions (Seoraksan National Park, Odaesan National Park, and Taebaeksan National Park), than toward the south (Juwangsan National Park, Woraksan National Park, and the Wangpicheon Conservation Area) (Figure 3). In previous studies, regardless of environmental conditions, microbiome diversity was shown to depend on resource availability, being high in food-rich areas and low in food-limited areas [20]. Also, studies have shown that the more diverse the gut microbiome is [21], the healthier it is, and therefore habitat conditions appear unfavorable toward the southern regions. Among the seven populations, Seoraksan showed the highest values in the indices of alpha diversity of gut microbiome, therefore, the goral population at this site seems healthier than those at the other sites. However, the populations in Juwangsan National Park, located in the southern margin, Woraksan National Park in the southwestern margin, and Wangpicheon Conservation Area in the eastern marginal region presented the lowest alpha diversity values. Based on these findings, more attention should be focused on the management and protection of long-tailed goral populations in more southern habitats, particularly in Juwangsan National Park, Woraksan National Park, and Wangpicheon Conservation Area.

In previous studies, the genetic diversity of Korean long-tailed goral populations, based on the analysis of partial mitochondrial DNA, was divided into two—north and south—geographical groups [3]. However, in the present gut microbiome research, conducted through beta diversity analysis, the populations were divided into three groups: by the PCoA Bray-Curtis distance, they were divided into (1) the Seoraksan National Park and Samcheock Area group, (2) the Wangpicheon Conservation Area group, (3) the Juwangsan National Park and Odaesan National Park group, and by PLS-DA with maximum distance, it was divided into (1) the Seoraksan National Park group, (2) the Samcheok Area group, and (3) the Wangpicheon Conservation Area group (Figure 4). Among the seven regions examined in our study, those with a self-sustainable population comprising at least one hundred individual gorals were Seoraksan National Park, Odaesan National Park, Wangpicheon Conservation Area, and Woraksan National Park. The maximum distance method analysis of beta diversity for the intestinal microbiota revealed that the Woraksan National Park—despite having a self-sustainable population—could not be divided into an independent group and belonged to the Wangpicheon Conservation Area. This seems to be due to the reintroduction of rescued individuals from several different populations. However, the verification of this hypothesis needs more detailed research conducted on a large number of samples. Although the region is not designated as a conservation or protected area, the Samcheok goral population appeared as an independent group and, together with the Seoraksan and Wangpicheon Conservation Area, Samcheok has an already sustainable population size. An increasing effort is needed to conduct detailed research studies on how to manage these populations, trace changes in them, and regulate the coexistence of the animals and the human population living in nearby areas.

### 4.2. Home Range, Dispersal, and Migration

Wild animals have three moving behaviors related to their habitat: staying within a certain range of an area (home range), scattering out of the herd due to competition (dispersal), or moving out of their home range (migration) [22]. Several studies on gorals investigating the seasonal and monthly home range, altitude changes, and habitat use have been conducted using GPS collars, but dispersal and migration in gorals has not been studied yet [23,24,25,26,27]. However, studies of these last two factors have been conducted on other species related to gorals. For example, it was shown that in male red deer (*Cervus elaphus*), 45% of the population migrated, and young males migrated up to 65 km [28]. Sika deer (*Cervus nippon*) migrated between their summer and winter habitats and traveled up to 31.9 km [29]. In the case of the European roe deer (*Capreolus capreolus*), 40% of the population migrated from the summer highlands to the winter lowlands over a distance of 12.0 ± 6.2 km [30], and males dispersed as one- or two-year-olds, or remained philopatric [31]. It should be noted that these studies investigated dispersal and migration within the same region or population, and that no studies of dispersal and migration between regions or populations have been conducted.

### 4.3. Site-Relational Network Analysis and Regional Migration

In the present study, we used site-relational networks with social network analysis and Animal Social Network Repositories, which are valuable tools to understand the patterns, evolution, and consequences of sociality [32]. We used network analysis because it requires data accumulation, and the biodiversity of the abundant microbial species constituting the gut microbiome can be effectively used as metadata [33]. So, for the first time, we have applied gut microbiome metadata to the long-tailed gorals, and we have analyzed migration patterns between regions or between populations (regional migration) in relation to diversity and composition changes of the gut microbiome.

The results of this network analysis showed that active migrations of goral populations occur between regions (Figure 5), even though the Samcheok Area is located more than 100 km away from Seoraksan National Park (Figure 1). Based on the analysis, these two regions are the most closely related (Figure 5), providing evidence that north-to-south migration occurred in the past. The present metadata analysis of the gut microbiome revealed that the Samcheok population is closest to the Seoraksan population, which is in contrast with a previous study suggesting that the Samcheok population was not included in the Seoraksan population [34]. The other populations were closely related to the Wangpicheon population. The site-relational network results obtained from the metadata analysis of the gut microbiome of long-tailed gorals have shown that a large migration flow occurred from north to south between populations, while a small migration flow took place in the opposite direction in a few cases. Each region showed a highly complex migration relationship. Long-tailed gorals seem to be actively migrating between the Samcheok Area and the Wangpicheon Conservation Area. In Juwangsan National Park, which is located at the southern edge the of distribution boundary of this species, gorals were discovered a few years ago and are known as a new population of small individual gorals. The connection between the Juwangsan National Park and Odaesan National Park populations, and the population of Samcheok Area (Figure 5 JW1, JW2), which is considerably distant, is explained by the fact that there was a migration of long-tailed gorals between Odaesan National Park and the Samcheok Area. It seems that a part of the individuals migrated to Juwangsan National Park. Woraksan National Park is the first reintroduction area used for the long-tailed goral restoration project, and here dozens of individuals rescued from other areas have been released. The long-tailed gorals of Woraksan National Park were diversely linked with the populations of Seoraksan National Park, Odaesan National Park, Wangpicheon Conservation Area, and the Samcheok Area. These results contrast with previous studies, which showed low connectivity between goral populations from two different regions [35]. Our metadata analysis of the gut microbiome of long-tailed gorals could adequately explain the variation in the diversity of populations and geographical migration between populations.

## 5. Conclusions

Although a large number of ecological studies exist on the long-tailed goral, the relationships or differences between populations in South Korea—and especially the possibility of migration—remained unclear. In this study we have used a gut microbiome metadata for network analysis. The alpha diversity, beta-diversity, and network analyses of the microbiome produced three different results. First, it was revealed that the Seoraksan population is the most stable and is a major migrating population. Second, there are three separate populations (Seoraksan, Samcheok, and Wangpicheon). Third, based on the network analysis, it appears that active migration occurs between populations, along a north-to-south migration route, and, occasionally, a few individual gorals move from south to north. Although Samcheok was not a designated as a protected area, this population was closely related to the Seoraksan population, and represented an independent group with sustainable individual gorals.

Overall, the present metadata analysis of the gut microbiome of long-tailed gorals could adequately explain the variation in the diversity of populations and geographical migration between populations.

## Data Availability

The microbiota data used in this study are available at Bioproject PRJNA739827.

## Supplementary Materials

The following are available online at https://www.mdpi.com/article/10.3390/microorganisms9092002/s1. Table S1: The composition percentage of five phyla of gut microbiome for each region. Figure S1: Statistical comparisons of abundances for phyla (Wilcoxon rank sum tests). Figure S2: Statistical comparisons of abundances for genera (Wilcoxon rank sum tests).
