# Supplementary material for: Geographical Relationships between Long-Tailed Goral (Naemorhedus caudatus) Populations Based on Gut Microbiome Analysis
Source: Microorganisms. 2021 Sep 21;9(9):2002. doi: 10.3390/microorganisms9092002 (PMC8468579; doi:10.3390/microorganisms9092002)
Supplement: Supplementary file 1 [file microorganisms-09-02002-s001.zip › microorganisms-1377137-supplementary.pdf]

microorganisms

Supplementary materials

Table S1. The composition percentage of five phyla of gut microbiome for each region.

Phylum	JW	OD	SA	SC	TB	WA	WPC
Firmicutes	59.86	18.27	26.52	20.13	7.40	41.04	13.53
Actinobacteriota	23.99	12.10	18.80	28.82	45.53	17.47	29.86
Bacteroidota	7.80	40.37	16.70	35.35	22.94	8.55	28.69
Proteobacteria	0.00	23.29	31.88	12.75	13.15	25.78	25.27
Euryarchaeota	8.35	0.69	2.17	0.63	5.29	0.29	0.25

Abbreviations: JW = Juwangsan National Park; OD = Odaesan National Park; SC = Samcheok Area; SA = Seoraksan National Park; TB = Taebaeksan National Park; WPC = Wangpicheon Conservation Area; WA = Woraksan National Park.

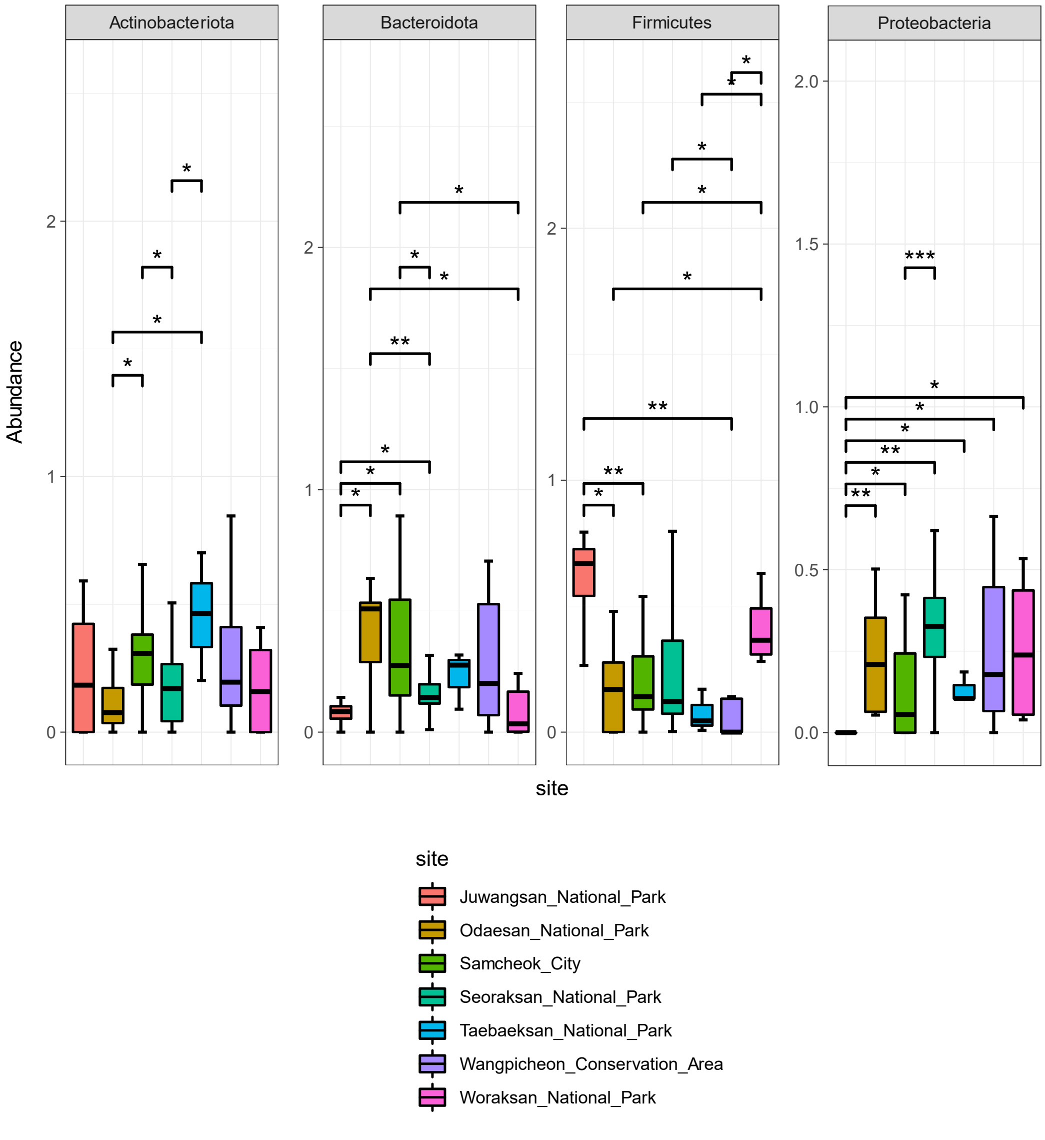

Figure S1. Statistical comparisons of abundances for phyla (Wilcoxon rank sum tests). * $P < 0.05$, ** $P < 0.01$, *** $P < 0.001$.

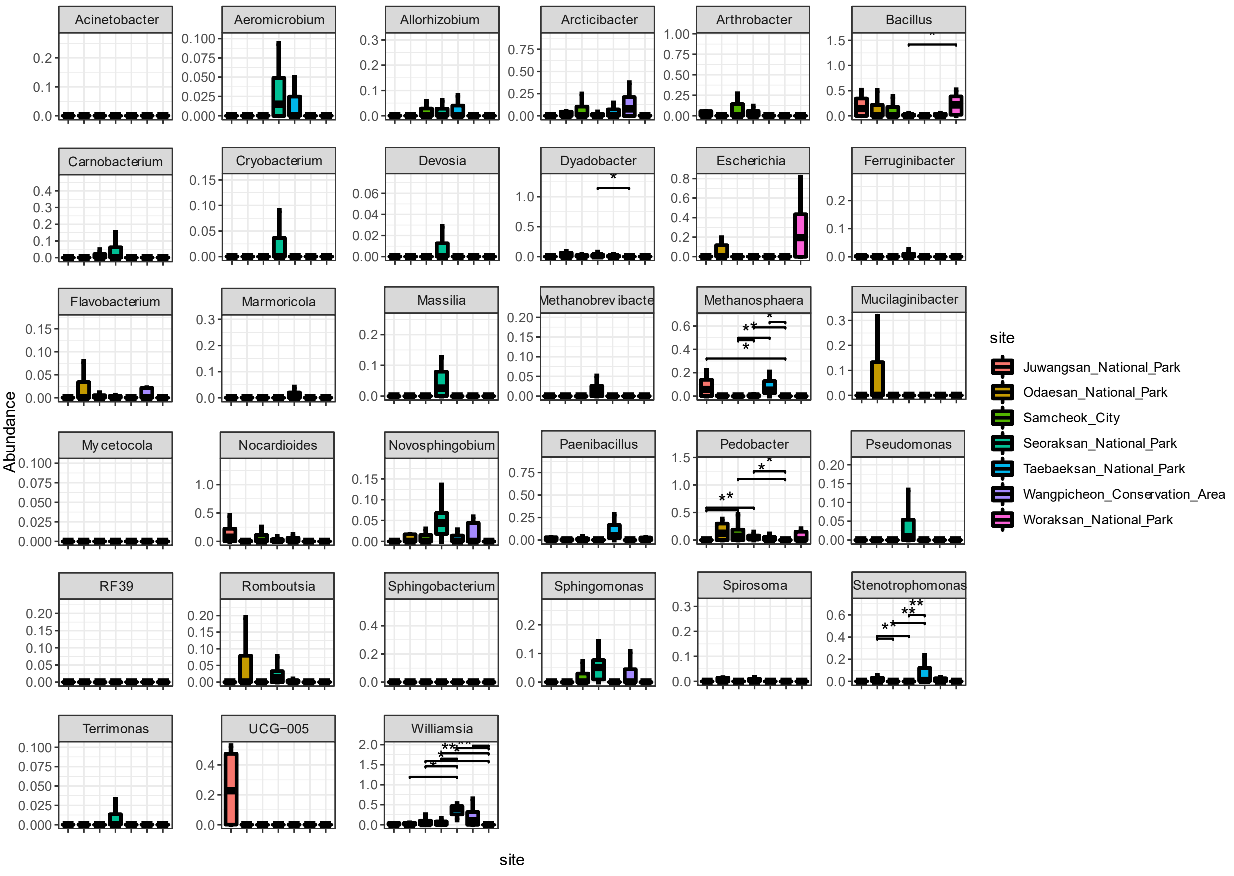

Figure S2. Statistical comparisons of abundances for genera (Wilcoxon rank sum tests). * $P < 0.05$, ** $P < 0.01$.
